# The CD-loop of PAI-2 (SERPINB2) is redundant in the targeting, inhibition and clearance of cell surface uPA activity

**DOI:** 10.1186/1472-6750-9-43

**Published:** 2009-05-14

**Authors:** Blake J Cochran, Lakshitha P Gunawardhana, Kara L Vine, Jodi A Lee, Sergei Lobov, Marie Ranson

**Affiliations:** 1 School of Biological Sciences, University of Wollongong, NSW, 2522, Australia; 2 Current address: Department of Respiratory and Sleep Medicine, Hunter Medical Research Institute, John Hunter Hospital, Newcastle, NSW, 2310, Australia

## Abstract

**Background:**

Plasminogen activator inhibitor type-2 (PAI-2, SERPINB2) is an irreversible, specific inhibitor of the urokinase plasminogen activator (uPA). Since overexpression of uPA at the surface of cancer cells is linked to malignancy, targeting of uPA by exogenous recombinant PAI-2 has been proposed as the basis of potential cancer therapies. To this end, reproducible yields of high purity protein that maintains this targeting ability is required. Herein we validate the use *in vitro *of recombinant 6 × His-tagged-PAI-2 lacking the intrahelical loop between C and D alpha-helices (PAI-2 ΔCD-loop) for these purposes.

**Results:**

We show that PAI-2 ΔCD-loop expressed and purified from the pQE9 vector system presents an easier purification target than the previously used pET15b system. Additionally, PAI-2 ΔCD-loop gave both higher yield and purity than wild-type PAI-2 expressed and purified under identical conditions. Importantly, absence of the CD-loop had no impact on the inhibition of both solution phase and cell surface uPA or on the clearance of receptor bound uPA from the cell surface. Furthermore, uPA:PAI-2 ΔCD-loop complexes had similar binding kinetics (K_D _~5 nM) with the endocytosis receptor Very Low Density Lipoprotein Receptor (VLDLR) to that previously published for uPA:PAI-2 complexes.

**Conclusion:**

We demonstrate that the CD-loop is redundant for the purposes of cellular uPA inhibition and cell surface clearance (endocytosis) and is thus suitable for the development of anti-uPA targeted cancer therapeutics.

## Background

Plasminogen activator inhibitor type-2 (PAI-2) is a clade B serine protease inhibitor (SERPIN) that is found as both a 60 kDa glycoprotein and a non-glycosylated 47 kDa form [1]. Both forms efficiently inhibit soluble or receptor-bound urokinase plasminogen activator (uPA) [1,2] by the classical serpin inhibitory mechanism resulting in irreversible inhibition of the enzyme [3]. The majority of expressed PAI-2 is not secreted and this may be linked to an inefficient, mildly hydrophobic internal signal peptide [4,5]. Thus, whilst PAI-2 levels in plasma are normally too low to be detected, in conditions such as pregnancy, some myelomonocytic leukemias and in inflammatory tissue, PAI-2 is consistently detected in plasma and other body fluids as both a glycoprotein and in the 47 kDa form [5-9]. This suggests a role for PAI-2 in extracellular protease inhibition *in vivo*.

We have previously shown that exogenous PAI-2 efficiently inhibits cell surface uPA receptor (uPAR)-bound uPA leading to the rapid clearance of the inhibited complex from the cell surface via receptor mediated endocytosis [2]. This involves interactions with endocytosis receptors of the Low Density Lipoprotein receptor (LDLR) family leading to delivery of uPAR/uPA/PAI-2 to endosomes and lysosomes [2,10,11]. Tumour overexpression of uPA/uPAR and the related uPA inhibitor PAI-1 (SERPINE1) strongly correlates to metastatic potential [12-16] and poor patient prognosis [17-20], but the presence of PAI-2 is associated with benign tumours and increased, relapse-free survival [9]. As such, we proposed that the ability of PAI-2 to remove cell surface uPA and hence proteolytic activity, without activation of the pro-mitogenic/motogenic signalling pathways associated with PAI-1 [9,11], accounts for the differential prognosis seen for PAI-2 versus PAI-1 [9-11].

Therefore, the ability of PAI-2 to specifically target uPA and hence tumour cells without interacting with components of the ECM or modifying other cellular behaviours that may promote tumour cell behaviour (unlike PAI-1) [11], supports the use of exogenous PAI-2 as the basis of uPA targeted cancer treatments. Promising results using bismuth-213 labelled PAI-2 have been obtained in a number of *in vitro*, *in vivo *and preclinical evaluations which show clear cell targeting specificity and tumour efficacy with minimal side effects in relevant animal models [21-27]. These studies used full length wild-type PAI-2, but it may be possible to utilise smaller, more easily producible PAI-2 constructs. This would require validation in terms of its extracellular uPA inhibitory and clearance functions.

Previous studies have reported the purification of PAI-2 from placenta [28], cultured human monocytes [29], transfected CHO cells [30,31], baculovirus infected insect larvae [32], yeast [33] and *Escherichia coli *[30,34-43]. Methods of PAI-2 expression in *E. coli *have generally utilised a one or two step purification procedure, usually involving metal affinity chromatography and/or ion exchange chromatography. The shift in the literature towards affinity tag based systems for the production of recombinant PAI-2 constructs [34-39] allows for the purification of PAI-2 under milder, native conditions and avoidance of denaturation/renaturation [35] or extreme pH treatment [30] as used previously. The presence of an *N*-terminal 6 × His-tag has previously been shown to have no significant impact on the uPA inhibitory activity of PAI-2 [36]. Generally, His-tags are believed to have no effect on overall protein structure [44].

An issue associated with the purification of recombinant wild-type PAI-2 is that PAI-2 contains a 33 amino acid intrahelical loop between alpha helices C and D (known as the CD-loop) which is accessible for cleavage in both *E. coli *or mammalian expression systems [34]. This results in two fractions of recombinant PAI-2 which retain inhibitory activity but require additional purification steps such as ion-exchange chromatography [34]. Di Giusto *et al*. [38] showed that 6 × His-tagged PAI-2 lacking the CD-loop (termed PAI-2 ΔCD-loop) can be purified with a one-step procedure and exhibited identical soluble phase uPA inhibitory activity.

The functionality of the CD-loop has been described primarily in an intracellular context and remains somewhat controversial [9]. The CD-loop is involved in transglutaminase mediated cross-linking to cellular and ECM proteins [43,45], although the functional significance of this cross-linking is unknown. Interestingly, cross-linked PAI-2 maintains uPA inhibitory activity. The CD-loop is believed to be highly mobile [34] and as such the crystal structure of PAI-2 has only been resolved for a CD-loop deletion mutant [42].

Altogether, this suggests that PAI-2 ΔCD-loop, being easy to produce and purify in addition to retaining uPA inhibitory activity, could be used as an exogenous uPA targeting substitute for wild-type PAI-2 in a therapeutic setting. To this end, we compared the expression and purification of 6 × His-tagged wild type PAI-2 and PAI-2 ΔCD-loop and show for the first time that the cell bound uPA inhibitory activity and rapid clearance of uPAR/uPA by PAI-2 is not compromised by the 6 × His-tagged form lacking the CD-loop.

## Results and discussion

### Expression and purification of PAI-2 ΔCD-loop

**Figure 1:**
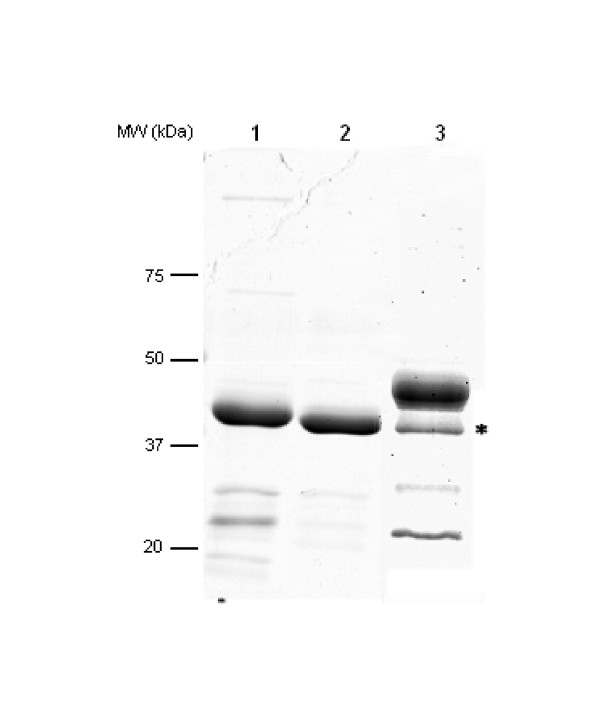
**SDS-PAGE analysis of highly enriched recombinant PAI-2 proteins**. 10 μg of total protein from PAI-2 ΔCD-loop purified from either pET15b (lane 1) or pQE9 (lane 2), or wild-type PAI-2 from pQE9 (lane 3) were fractionated by a 10% SDS-PAGE under reducing conditions. The slight difference in size between PAI-2 ΔCD-loop from pET15b and pQE9 is due to differences in tag/linker length. * marks cleaved PAI-2 in wild-type population as mentioned in text.

**Figure 2:**
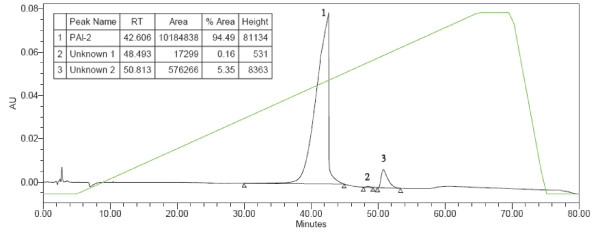
**Quantitative analysis of the purification of PAI-2 ΔCD-loop from pQE9**. Protein (100 μL) was injected onto a cation exchange column and eluted using a linear NaCl gradient (0–1 M) at 1 mL/min. PAI-2 ΔCD-loop was detected at 280 nm with a RT of 42.606 min. Integration of the peak corresponding to PAI-2 ΔCD-loop measured the purity at 94.5%. The green line indicates the relative amount of buffer B (MES pH 5.0, 1 M NaCl) to buffer A (MES pH 5.0). Insert: Relative retention times hatand purity as calculated from AUC using Empower Pro V2 (Waters) software. RT = retention time.

Expression and purification of PAI-2 ΔCD-loop from both the pET15b and pQE9 vector systems yielded a product migrating as a major band at 45 kDa (Figure 1). This corresponds to the expected molecular weight of PAI-2 ΔCD-loop and was confirmed via western blotting with a monoclonal antibody to PAI-2 (data not shown). The PAI-2 ΔCD-loop from the pQE9 system contained fewer impurities (seen as lower molecular weight bands; Figure 1) compared to PAI-2 ΔCD-loop from pET15b expressed and purified under similar conditions (Figure 1, compare lanes 1 and 2). The abundance of PAI-2 ΔCD-loop expressed and purified from the pQE9 versus the pET15b system was found to be ~95% pure compared to ~74%, respectively, as measured by densitometry. The purity of PAI-2 ΔCD-loop was confirmed via HPLC, with integration of the elution profile giving PAI-2 ΔCD-loop an abundance of ~95% (Figure 2). The expression and purification of PAI-2 ΔCD-loop using the pQE9 system regularly achieved yields between 10 and 15 mg/L of culture. Wild-type PAI-2 expressed and purified using the pQE9 system was less pure (Figure 1, compare lanes 1 and 3) and of lower yield (~5 mg/L) compared to PAI-2 ΔCD-loop. When visualised by SDS-PAGE under reducing conditions, wild-type PAI-2 yielded two bands that were identified as PAI-2 via western blotting (data not shown). This confirms our previous observation that a portion of wild-type PAI-2 is cleaved in the CD-loop [34], which would require further purification steps to obtain a homogenous protein population.

### Electrospray Ionisation Mass Spectrometry (ESI-MS)

**Figure 3:**
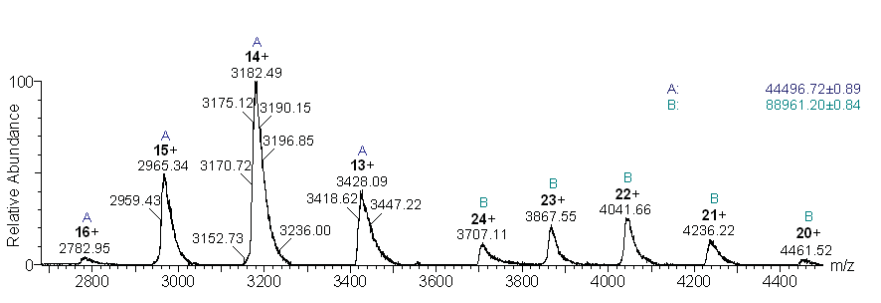
**A positive ion ESI-MS of PAI-2 ΔCD-loop from pQE9 in 10 mM ammonium acetate (pH 6.8) containing 0.1% formic acid**. The m/z spectrum shows a Gaussian-type distribution of multiply charged ions ranging from m/z 2700 – 4500. Charge states are indicated. The monomeric (**A**) and dimeric (**B**) forms of the protein gave a measured molecular mass of 44496 Da (± 0.89) and 88961 Da (± 0.84), respectively. Mass was calculated using MassLynx MS software (Waters) and caesium iodide was used for external calibration.

The mass spectrometry data presented here is the first spectrum of any PAI-2 construct to be published. The ESI-MS spectrum of PAI-2 ΔCD-loop (Figure 3) shows a pattern of multiply charged ions (13^+ ^to 16^+^) in the *m*/*z *range of 2700–3500 consistent for the 45 kDa monomeric species. The calculated molecular weight of 44496.72 Da is in agreement with the theoretical monoisotopic molecular weight of 44480.16 Da. In addition, five higher charge states (20^+ ^to 24^+^) from *m/z *3,700 – 4,500 representing an 89 kDa dimeric species were also observed. Lobov *et al*. [34] showed that whilst less polymergenic than wild-type PAI-2, PAI-2 ΔCD-loop was still able to form dimers and higher order aggregates.

### The absence of the CD-loop does not affect the solution phase or cell surface uPA activity inhibitory function of PAI-2

**Figure 4:**
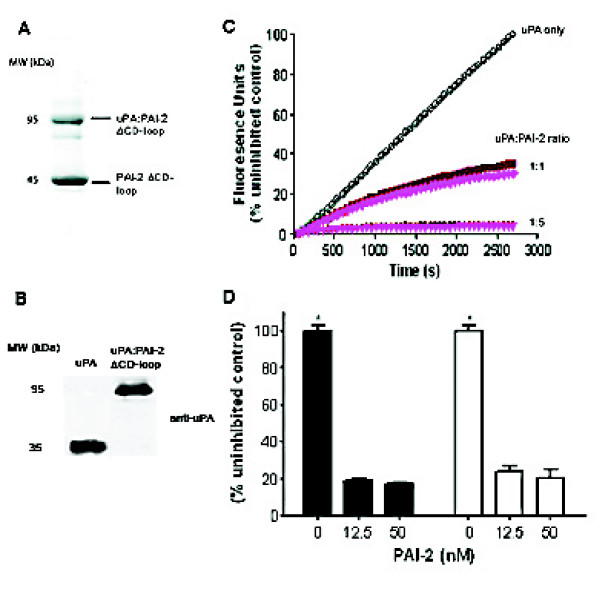
**PAI-2 ΔCD-loop efficiently inhibits both solution phase and cell bound uPA activity**. **A**. PAI-2 ΔCD-loop is able to form SDS stable complexes with uPA. PAI-2 ΔCD-loop was incubated in a 2:1 molar ratio with HMW-uPA for 30 min and analysed by a 10% SDS-PAGE under reducing conditions. **B**. Immunoblot analysis of complex formation between uPA and PAI-2 ΔCD-loop using a monoclonal antibody to the A-chain of uPA (~30 kDa), indicating that no residual uPA remains in the sample. **C**. Kinetic inhibition curves for wild-type PAI-2 (dark red) versus PAI-2 ΔCD-loop (bright pink) against HMW-uPA in solution. The uPA fluorogenic substrate was briefly pre-incubated with the PAI-2 forms or buffer alone (open circles) and the assays initiated by the addition of HMW-uPA. Fluorescence units were converted to a percentage of the maximal (uninhibited) uPA activity. Values shown are the means of triplicate determinations. Errors (<10%) are not shown for clarity of presentation. **D**. U937 cells were incubated with uPA fluorogenic substrate in the absence or presence of wild-type PAI-2 (filled bars) or PAI-2 ΔCD-loop (open bars) for 1 h at 37°C. Fluorescence units were converted to a percentage of the maximal (uninhibited) uPA activity. Values shown are means ± SEM (n = 3). *p < 0.001 compared to inhibited samples for both PAI-2 forms.

To ensure that the PAI-2 ΔCD-loop construct generated in this study retained uPA inhibitory activity, excess PAI-2 ΔCD-loop was incubated with uPA and analysed by SDS-PAGE under reducing conditions (Figure 4A). A band at ~95 kDa was observed corresponding to covalently complexed uPA:PAI-2 ΔCD-loop. Additionally, western blotting of this complex formation using a monoclonal antibody to uPA showed no free uPA remained after incubation (Figure 4B). PAI-2 ΔCD-loop and wild-type PAI-2 gave super-imposable kinetic inhibition curves for HMW-uPA in solution (Figure 4C), confirming that the absence of the CD-loop does not affect uPA inhibitory activity of PAI-2. Di Giusto *et al*. [38] showed that His-tagged PAI-2 ΔCD-loop had the same second order rate constant (2.40 × 10^6^M^-1 ^s^-1^) as the published wild-type non-recombinant form. Moreover, we show that the inhibitory activity of both variants was also identical towards cell surface receptor bound uPA as there were no significant differences seen between the two PAI-2 forms (Figure 4D). This indicates that the absence of the CD-loop is also redundant in the more physiological setting of the cell surface. This is to be expected as the majority of serpin sequence deviation occurs in the loops joining secondary structures [46]. Moreover, the intrahelical CD-loop is a unique feature of clade B serpins [47] which is absent (and hence not required for function) in inhibitory serpins of all other clades.

### Removal of the CD-loop does not affect the clearance of uPA from the cell surface

**Figure 5:**
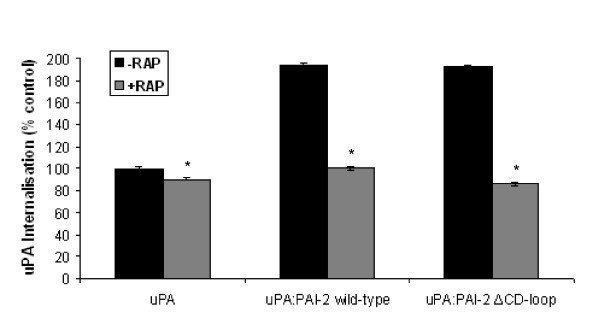
**Cellular internalisation of uPA is equivalent between wild-type PAI-2 and PAI-2 ΔCD-loop as determined by flow cytometry**. MCF-7 cells were incubated with 10 nM uPA:Alexa_488_, either alone or in complex with wild-type PAI-2 or PAI-2 ΔCD-loop for 1 h at 37°C. Any surface bound uPA:Alexa_488 _remaining was quenched by incubation with 4 μg/mL anti-Alexa_488 _polyclonal antibody for 30 min prior to analysis by dual colour flow cytometry. Values shown are means ± SEM (n = 3) as a percentage of uPA only treatment.*p < 0.005 compared to internalisation in the absence of RAP for each treatment.

Preliminary dot-blot analysis suggested that removal of the CD-loop of PAI-2 has no impact on the interaction between uPA complexed PAI-2 and members of the LDLR family [10]. Surface plasmon resonance was used to measure the affinity of uPA complexed with PAI-2 ΔCD-loop or wild-type PAI-2 for the endocytosis receptor VLDLR. The K_D _of both uPA:PAI-2 ΔCD-loop and uPA:wild-type PAI-2 for VLDLR was found to be ~5 nM (Table 1) and these interaction best fit a 1:1 binding model. Additionally, no interaction was observed between VLDLR and either PAI-2 ΔCD-loop or wild-type PAI-2 alone (Table 1). These findings are in agreement with those of Croucher *et al*. [11], which was determined using non-tagged, wild-type PAI-2. Additionally, when complexed with uPA, both wild-type PAI-2 and PAI-2 ΔCD-loop lead to an almost two-fold increase in the amount of uPA endocytosed by MCF-7 cells relative those treated with uPA alone (Figure 5). Importantly, there was no significant difference between cells treated with uPA:wild-type PAI-2 and uPA:PAI-2 ΔCD-loop. Pre-treatment of cells with the LDLR antagonist RAP significantly reduced the amount of uPA internalised in all treatments (Figure 5). This also confirms the role of VLDLR in endocytosis, as it is the only LDLR family member of relevance on MCF-7 cells [11].

**Table 1:** Kinetic parameters of the interaction between PAI-2 ΔCD-loop or wild-type PAI-2 and VLDLR, both alone and in complex with uPA.

**Analyte**	**Binding Model**	**k_a_** **(M^-1 ^s^-1^)**	**k_d_** **(s^-1^)**	**K_D_** **(nM)**	**χ^2^**
**PAI-2 wild-type**	No binding	-	-	-	-

**uPA:PAI-2 wild-type**	1:1	3.75 × 10^5^ (± 1.1 × 10^5^)	1.44 × 10^-3^ (± 0.39 × 10^-3^)	4.81 (± 0.64)	4.71

**PAI-2 ΔCD-loop**	No binding	-	-	-	-

**uPA:PAI-2 ΔCD-loop**	1:1	4.16 × 10^5^ (± 1.8 × 10^5^)	1.36 × 10^-3^ (± 0.23 × 10^-3^)	4.34 (± 0.56)	3.28

Values were determined using surface plasmon resonance. Binding data was fitted using the BIAevaluation 4.0 software. The binding model chosen represents that with the lowest χ^2 ^value. (Values are the average ± SEM, n = 3).

## Conclusion

This study shows that the CD-loop of PAI-2 plays no role in the inhibition and clearance of cell surface uPA *in vitro*. Additionally, we confirm previous findings indicating that the use of PAI-2 ΔCD-loop is advantageous compared to wild-type PAI-2 due to superior purity and yield of the recombinant protein under identical conditions. As such, PAI-2 ΔCD-loop appears to present a desirable basis for the development of PAI-2 based uPA targeted cancer therapies. Furthermore, tumour uptake increases as protein size is decreased [48], suggesting that this shortened but fully active form of PAI-2 may not only be simpler to express and purify than wild-type PAI-2, but also exhibit a favourable pharmacokinetic profile.

## Methods

### Materials

The expression vectors pET15b/PAI-2 wild-type and pET15b/PAI-2 ΔCD-loop were a kind gift from Prof. T Ny (Umea University, Sweden); pQE9 vector and M15 [pREP4] *E. coli *obtained from QIAgen; Oligonucleotides from Sigma-Genosys; phorbyl myristate acetate (PMA) from Sigma-Aldrich; Alexa_488 _labelling kit, Alexa_488 _polyclonal antibody and BL21 Star (DE3) *E. coli *from Invitrogen; IPTG from Applichem; Monoclonal antibodies against human uPA (#394) and human PAI-2 (#3750), and high molecular weight (HMW)-uPA from American Diagnostica; Ampicillin and kanamycin from Amresco; TALON metal affinity resin from Clontech; *Bam*HI restriction enzyme, shrimp alkaline phosphatase and T4 DNA ligase from Fermentas; CM5 BIAcore chip and PD-10 desalting columns from GE BioSciences; Recombinant VLDLR ligand binding region was a gift from D Blaas (Medical University of Vienna, Austria); Z-Gly-Gly-Arg-AMC from Calbiochem; Wild-type PAI-2 from PAI-2 Pty Ltd; Pfu HS Fusion II DNA polymerase from Stratagene.

### Generation of pQE9/PAI-2 constructs

Wild-type PAI-2 and PAI-2 ΔCD-loop cDNAs lacking the 3' untranslated region were amplified from pET15b using Pfu HS Fusion II DNA polymerase and the following primers: 5'-GCGCGGATCCCTCGAGGATCTTTGTGTGGC-3' (forward) and 5'-GCGCGGATCCTTAGGGTGAGGAAAATCTGCCG-3' (reverse). Primers contained *Bam*HI sites to allow for ligation into pQE9. Both vector and insert were digested with *Bam*HI, linear pQE9 de-phosphorylated using shrimp alkaline phosphatase and the insert ligated into the vector using T4 DNA ligase. Successful insertion and sequence integrity were confirmed using restriction digestion and DNA sequencing.

### Expression and purification of PAI-2

Purified pET15b/PAI-2 ΔCD-loop, pQE9/PAI-2 ΔCD-loop and pQE9/PAI-2 wild-type vectors were transformed into chemically competent BL21 Star (DE3) and M15 [pREP4] cells, respectively, using standard methods. Cells were cultured overnight at 37°C with shaking in LB containing 100 μg/mL ampicillin and 25 μg/mL kanamycin (only for M15 [pREP4] cells). A 20 mL aliquot of this starter culture was added to 1 L Z-broth (LB with the addition of 1 g/L glucose and 0.49 g/L CaCl_2_.2H_2_O) and grown to an OD_600 _of ~0.6. Expression of PAI-2 ΔCD-loop was induced by the addition of 0.5 mM IPTG and the culture incubated for a further 4 h. Cells were collected by centrifugation at 10,000 *g *for 10 min. Pelleted cells were resuspended in 15 mL of ice-cold loading buffer (50 mM NaH_2_PO_4_, 300 mM NaCl, pH 7.0, containing 5 mM imidazole for pET15b expressed PAI-2 ΔCD-loop) and lysed using a French press (Thermo, USA). The cell lysate (~20 mL) was then incubated with 2.5 μg of DNase for 30 min on ice and cell debris pelleted by centrifugation at 17,000 *g *for 30 min. The supernatant was loaded onto equilibrated TALON metal affinity resin at a rate of 1 mL/min. Unbound proteins were removed using 10 column volumes of loading buffer followed by 10 column volumes of wash buffer containing optimised imidazole concentrations (data not shown) (50 mM NaH_2_PO_4_, 300 mM NaCl, 5 mM imidazole for pQE9, 15 mM for pET15b, pH 7.0). Bound protein was eluted from the column using elution buffer (50 mM NaH_2_PO_4_, 300 mM NaCl, 150 mM imidazole, pH 7.0). Purification was analysed using SDS-PAGE under reducing conditions and the successful isolation of PAI-2 ΔCD-loop determined by western blotting using a monoclonal antibody against PAI-2. Samples were buffer exchanged into phosphate buffer (50 mM NaH_2_PO_4_, 300 mM NaCl, pH 7.0) to remove imidazole for subsequent experiments.

### Cation-exchange high performance liquid chromatography (HPLC)

The purity of PAI-2 ΔCD-loop expressed and purified from pQE9 was further determined by ion exchange chromatography on a BioSuite SP 10 μm, C × C (7.5 cm × 75 mm) cation exchange semi-preperative column (Waters, UK). The column was pre-equilibrated with approximately 10 column volumes of 20 mM 2-(*N*-morpholino)ethanesulfonic acid (MES, pH 5.0). PAI-2 ΔCD-loop (100 μL, ~1 mg/mL) was then loaded and eluted with a linear (curve 6) 0.5 M NaCl salt gradient at 1 mL/min. The data was collected on a photodiode array (PDA) at 280 nm and percent purity determined using Empower Pro V2 (Waters, UK) software.

### Electrospray ionisation mass spectrometry (ESI-MS)

A positive ion mass spectrum of PAI-2 ΔCD-loop expressed and purified from pQE9 was acquired on a quadrupole time of flight spectrometer (Q-TOF-MS) (Micromass Q-TOF Ultima, Waters, UK) fitted with a Z-spray ionisation source. A sample of freshly prepared protein in PBS pH 7.4 was exchanged into 10 mM ammonium acetate buffer (pH 6.8) containing 0.1% formic acid and made up to a final concentration of 10 μM. The protein was then injected into the Q-TOF Ultima mass spectrometer (20 μL) and the mass spectrum acquired with a capillary voltage of 2.6 kV, cone voltage of 50 V, source block temperature of 40°C, and a resolution power of 5000 Hz. Ceasium iodide was used for external calibration. The mass spectrum data is presented as raw data, on an *m*/*z *scale. Mass was calculated using MassLynx MS software (Waters).

### Fluorogenic uPA activity assay

Several substrate and uPA concentrations were used to find the optimum range and to set the gain on a Fluorostar Optima fluorescence plate reader (BMG Labtech, Germany). PAI-2 ΔCD-loop or wild-type PAI-2 were diluted in reaction buffer (20 mM Hepes, pH 7.6, 100 mM NaCl, 0.5 mM EDTA, 0.01% (v/v) Tween 20) and mixed with fluorogenic substrate, Z-Gly-Gly-Arg-AMC in 180 μL reaction buffer. After a brief preincubation at 37°C, HMW-uPA (final concentration 0.675 nM) was added to start the reaction and fluorescence emission measured immediately at 37°C. All assays were performed in triplicate and values corrected by subtracting the background (reaction buffer plus substrate only). For cell based assays, PMA treated U937 cells (used to enhance the expression of unoccupied uPAR [49]) were washed and preincubated with 50 nM HMW-uPA in binding buffer (Phenol red free Hanks buffered salt solution, pH 7.4, containing 1 mM CaCl_2_, 1 mM MgCl_2 _and 0.1% BSA) for 10 min to saturate uPAR. After 2 washes the cells were resuspended in binding buffer to give a final concentration of 5 × 10^5 ^cells/mL. To initiate the reaction 100 μL aliquots of the cell suspension were transferred to a 96-well fluor plate containing 100 μL of 0.5 mM substrate +/- PAI-2 in binding buffer pre-equilibrated to 37°C (final concentrations, 0.25 mM substrate, 12.5 or 50 nM PAI-2) and fluorescence emission measured. All assays were performed in triplicate and values corrected by subtracting background fluorescence [binding buffer plus substrate +/- PAI-2 (the presence of PAI-2 made no difference to background levels)]. Differences in uPA activity were tested for significance using an unpaired Student's t-test.

### Surface plasmon resonance

Surface plasmon resonance was used to determine the impact of the removal of the CD-loop and presence of the 6 × His-tag on the affinity of uPA:PAI-2 complexes for VLDLR, essentially as described by Croucher *et al*. [11]. VLDLR was immobilised on a CM5 BIAcore chip, according to the manufacture's instructions. Briefly, the chip was activated with a 1:1 mixture of 0.2 M *N*-ethyl-*N*'-(3-dimethylaminopropyl)carbodi-imide and 0.05 M *N*-hydroxysuccimide. VLDLR was coated onto the chip at 40 μg/mL in 10 mM sodium acetate (pH 3) to a level of ~2,000 response units. Unoccupied binding sites were blocked using 1 M ethanolamine, pH 8.5. Ligands were diluted into running buffer (10 mM Hepes, pH 7.4, 150 mM NaCl, 1 mM CaCl_2_, 0.005% Tween-20) before application to the BIAcore chip at 20 μL/min. Regeneration was achieved using 100 mM H_3_PO_4_. Data was analysed using BIAevaluation software (Version 4), using a blank cell as the reference cell.

### Internalisation assays

Internalisation assays were conducted essentially as previously described [2,10,11], using the MCF-7 cell line. Briefly, cells were grown for 48 h in 6 well plates to subconfluency, washed with phenol red free hanks buffered salt solution (pH 7.4, containing 1 mM CaCl_2_, 1 mM MgCl_2 _and 0.1% BSA), then incubated in binding buffer for 1 h at 37°C to allow for receptor recycling. To confirm LDLR specificity, cells were preincubated for 10 min with 200 nM RAP (receptor associated protein). Cells were incubated with 10 nM uPA:Alexa_488_, both alone and previously complexed with either wild-type PAI-2 or PAI-2 ΔCD-loop for 1 h at 37°C to allow for internalisation. After this incubation period, the cells were washed twice with ice cold binding buffer, harvested using 5 mM EDTA, washed again and resuspended in ice cold binding buffer containing 4 μg/mL Alexa_488 _quenching polyclonal antibody. After 30 min the cells were washed twice with ice-cold PBS and analysed by dual colour flow cytometry with propidium iodide to exclude non-viable cells as previously described [2]. Differences in the amount of uPA internalised between treatments were tested for significance using the unpaired Student's t-test.

## Abbreviations

AUC: Area under the curve; ECM: Extracellular matrix; HMW: High molecular weight; HPLC: High performance liquid chromatography; LDLR: Low density lipoprotein receptor; PAI-2: Plasminogen activator inhibitor type-2; PAI-1: Plasminogen activator inhibitor type-1; PMA: Phorbyl myristate acetate; RAP: Receptor associated protein; RT: Retention time; uPA: Urokinase plasminogen activator; uPAR: Urokinase plasminogen activator receptor; VLDLR: Very low density lipoprotein receptor.

## Authors' contributions

BC compiled and wrote the manuscript, carried out experiments and assisted in planning of the study. LG built the constructs used in this study and carried out initial experimental work. KV conducted experiments, contributed data and wrote sections of the manuscript. JL provided and analysed experimental data. SL was involved in the planning and supervision of the project and writing sections of the manuscript. MR was involved in the planning, funding and supervision of the study, writing of the manuscript and generating experimental data. All authors read and approved the final manuscript.
